# Hyaluronic Acid 35 kDa Protects against a Hyperosmotic, Formula Feeding Model of Necrotizing Enterocolitis

**DOI:** 10.3390/nu14091779

**Published:** 2022-04-24

**Authors:** Kathryn Burge, Jeffrey Eckert, Adam Wilson, MaJoi Trammell, Shiloh R. Lueschow, Steven J. McElroy, David Dyer, Hala Chaaban

**Affiliations:** 1 Department of Pediatrics, Division of Neonatology, University of Oklahoma Health Sciences Center, Oklahoma City, OK 73104, USA; kathryn-burge@ouhsc.edu (K.B.); jeffrey-eckert@ouhsc.edu (J.E.); adam-wilson@ouhsc.edu (A.W.); 2 Department of Microbiology and Immunology, University of Oklahoma Health Sciences Center, Oklahoma City, OK 73104, USA; majoi-trammell@ouhsc.edu (M.T.); david-dyer@ouhsc.edu (D.D.); 3 Department of Microbiology and Immunology, Stead Family Department of Pediatrics, University of Iowa, Iowa City, IA 52242, USA; shiloh-lueschow@uiowa.edu; 4 Department of Pediatrics, University of California Davis, Sacramento, CA 95817, USA; sjmcelroy@ucdavis.edu

**Keywords:** necrotizing enterocolitis, hyaluronic acid, prematurity, formula feeding, human milk, osmolality, microbiome

## Abstract

Necrotizing enterocolitis (NEC), an inflammatory disease of the intestine, is a common gastrointestinal emergency among preterm infants. Intestinal barrier dysfunction, hyperactivation of the premature immune system, and dysbiosis are thought to play major roles in the disease. Human milk (HM) is protective, but the mechanisms underpinning formula feeding as a risk factor in the development of NEC are incompletely understood. Hyaluronic acid 35 kDa (HA35), a bioactive glycosaminoglycan of HM, accelerates intestinal development in murine pups during homeostasis. In addition, HA35 prevents inflammation-induced tissue damage in pups subjected to murine NEC, incorporating Paneth cell dysfunction and dysbiosis. We hypothesized HA35 treatment would reduce histological injury and mortality in a secondary mouse model of NEC incorporating formula feeding. NEC-like injury was induced in 14-day mice by dithizone-induced disruption of Paneth cells and oral gavage of rodent milk substitute. Mortality and histological injury, serum and tissue cytokine levels, stool bacterial sequencing, and bulk RNA-Seq comparisons were analyzed. HA35 significantly reduced the severity of illness in this model, with a trend toward reduced mortality, while RNA-Seq analysis demonstrated HA35 upregulated genes associated with goblet cell function and innate immunity. Activation of these critical protective and reparative mechanisms of the small intestine likely play a role in the reduced pathology and enhanced survival trends of HA-treated pups subjected to intestinal inflammation in this secondary model of NEC, providing potentially interesting translational targets for the human preterm disease.

## 1. Introduction

Necrotizing enterocolitis (NEC), a devastating inflammatory condition of the intestine, affects up to 10% of premature infants born less than 1.5 kg [1], with a mortality rate exceeding 30% if surgery is required [2]. While the etiology of the disease is still unclear, intestinal dysbiosis, poor regulation of the intestinal barrier, and hyperactivation of the immature intestinal immune system are thought to play important roles [3,4,5].

Exclusive human milk (HM) feeding is known to protect against NEC [6,7], likely through the combined effects of bioactive components such as lactoferrin, oligosaccharides, and immunomodulators [8]. Hyaluronic acid (HA), a glycosaminoglycan composed of repeating β-d-glucuronic and *N*-acetyl-β-d-glucosamine disaccharides, is present in significantly higher amounts in HM compared with infant formula, particularly during the first postnatal month [9]. When degraded from the high molecular weights produced endogenously, the size of resulting HA fragments, in part, determines the physiological effects [10]. The pro- or anti-inflammatory properties of HA fragments also depend upon the physiological milieu in which they are deposited, as well as their mode of administration [11]. In the colon, HA35 (HA of an average molecular weight of 35 kDa, a HM HA mimic) induces the expression of murine antimicrobial β-defensins and strengthens the epithelial barrier [9,12]. Our prior work demonstrated that treatment with HA35 increased small intestinal crypt depth, villus length, and goblet and Paneth cell numbers during a period of 7 days of postnatal development [13]. HA treatment also remodeled the microbiome of mouse pups, increasing the prevalence of species associated with enhanced intestinal barrier function. Furthermore, the ileal transcriptome in these pups demonstrated HA-associated upregulation in genes critical to proliferation and epithelial survival. Our group also showed HA35 is protective in a mouse model (dithizone/*Klebsiella* [14]) of NEC utilizing dithizone-induced Paneth cell disruption and induction of dysbiosis through oral gavage of *Klebsiella pneumoniae* [15]. HA35 significantly reduced histological intestinal injury and mortality through a reduction in epithelial permeability, proinflammatory cytokine release, and bacterial translocation, as well as an increase in tight junction protein expression.

Formula feeding is a commonly identified risk factor for the development of NEC [16], but one not included in our prior modeling of the effects of HA35 on NEC development. Animal modeling has demonstrated that formula-induced intestinal inflammation may reproduce the type of dysbiosis associated with early NEC [17,18], rendering this risk factor particularly important for inclusion in translational animal models of the disease. Beyond lacking many of the bioactive components of HM, formula is thought to predispose infants to development of NEC due, in part, to increased osmolality [19,20] the ratio of solutes to solvent [21]. While preterm and term HM measures roughly 275 and 300 mOsm/kg H_2_O, respectively, formula ranges between 250 and 360 mOsm/kg H_2_O, depending upon brand [21]. This high solute concentration of formula can also be extrapolated to nutritionally fortified HM (360–415 mOsm/kg H_2_O), often required in the preterm population when aggressive postnatal growth is sought [21,22,23]. In addition to osmolality concerns, formula has been associated with intestinal ischemia and appears to induce microcirculatory changes in the intestinal mucosa [24,25].

Given the often-poor translational efficacy of therapies tested in a single in vivo model system [26], we sought to determine whether HA35 would be similarly effective in a secondary NEC model encompassing both dithizone-induced Paneth cell dysfunction and formula feeding (D/FF NEC model) [18]. As HA35 has been effective in multiple intestinal inflammatory conditions [12,15,27], we hypothesized that mortality, intestinal injury, and systemic inflammation would be similarly ameliorated by HA35 in the D/FF NEC model. Our intriguing data indicate that HA35 not only reduces the histological severity of NEC intestinal injury, but also increases the intestinal gene expression associated with mucosal protection and goblet cell function.

## 2. Materials and Methods

### 2.1. Formula Preparation

Rodent milk substitute (RMS) formula, adapted from Dvorak et al. [28], was prepared 24 h before the experiment. The composition of RMS is described in Table 1. The components of RMS included KH_2_PO_4_, MgSO_4_, FeSO_4_·7H_2_O, KI, NaF, Al_2_(SO_4_)_3_·18H_2_O (Fisher Scientific, Waltham, MA, USA), MnSO_4_, l-ascorbic acid sodium salt, riboflavin, niacin, pyridoxal hydrochloride (Fisher Scientific), myo-inositol (ThermoFisher, Waltham, MA, USA), NaCl, KCl, CaCO_3_, calcium lactate pentahydrate, evaporated milk (Great Value, Wal-Mart, Bentonville, AR, USA), bovine serum albumin, Teklad vitamin mix (#40060, Harlan Teklad, Madison, WI, USA), IntraLipid 20% intravenous fat emulsion, sunflower oil (ThermoFisher), ZnSO_4_, CuSO_4_, and ddH_2_O. All components were purchased from Sigma-Aldrich (St. Louis, MO, USA) unless otherwise indicated. Per 100 g, the macronutrient composition of this RMS was approximately 7.6 g protein, 11.4 g lipid, and 6.2 g carbohydrate, totaling 650 kJ energy, with an osmolality of 660 mOsm/kg H_2_O [28].

### 2.2. Mouse Model

The Institutional Animal Care and Use Committee (IACUC, Protocol #19–062-EF-CHI) of the University of Oklahoma Health Sciences Center approved all animal experiments, and experiments were performed according to guidelines stated in the Guide for the Care and Use of Laboratory Animals [29]. Timed pregnant CD-1 dams (Charles River Laboratories, Wilmington, MA, USA) were housed individually in sterilized, filter-top cages, subjected to a 12 h light/dark cycle, and provided ad libitum food and water. Pups were born vaginally and randomized at P6 (postnatal day of life 6) to one of the following groups: (1) Sham, (2) HA (HA alone), (3) formula (formula alone), (4) formula + HA, (5) NEC (dithizone + formula), or (6) NEC + HA. Beginning at P7, sodium hyaluronate (Lifecore Biomedical, Chaska, MN, USA) with an average molecular weight of 35 kDa was administered to pups in the NEC+HA, formula + HA, and HA alone groups by gavage at a concentration of 30 mg/kg body weight once daily for 7 days prior to induction of NEC. Pups not receiving HA35 were administered an equal volume of sterile water. Newborn 24-gauge blunt feeding needles (Cadence Science, Cranston, RI, USA) were used for oral gavage. Decisions on HA35 dosing level and duration were based on our previous studies [13,15]. Pups were weighed periodically during the 7-day pretreatment period. All pups were dam-fed prior to induction of NEC. On the morning of the experiment, pups were separated from dams and maintained in a temperature- and humidity-controlled chamber. NEC was induced using a combination of Paneth cell disruption and formula feeding [18]. P14 pups (intestinal development equivalent to that of a human at 22–24 weeks of gestation [30,31]) were gavaged 200 µL RMS (prepared as per Section 2.1) every 3 h for a total of four feeds (Table 2). Shams were removed from the dams only for gavage of an equivalent volume of sterile saline. One hour following the first formula feed, pups were injected intraperitoneally with dithizone (33 mg/kg; Sigma-Aldrich) diluted in ethanol/ammonium hydroxide, inducing Paneth cell dysfunction via zinc chelation [32]. Pups were monitored for clinical illness and mortality for 13 h after the first formula feed, at which time tissues were harvested following humane euthanasia. This study, in total, utilized six different litters, and litter-to-litter variability was minimized via the use of pups from at least three different litters in each experiment. All controls were littermates of animals randomized to experimental groups.

### 2.3. Histology and Injury Scoring

Tissues from the distal ileum were preserved in 10% buffered formalin for microscopic evaluation of intestinal injury. Tissues were paraffin-embedded, and serial sections of 5 µm were cut. The tissues were deparaffinized, rehydrated, and stained with hematoxylin and eosin (H&E). The severity of the gut injury was assessed by two investigators blinded to treatment, using a modified four-point scoring system [33,34]: Grade 0—normal, intact mucosa; Grade 1—mild development of subepithelial Gruenhagen’s space, vacuolization of villi, or subepithelial lifting of the lamina propria or tips of villi; Grade 2—moderate epithelial lifting and vacuolization equivalent to at least half of villi; Grade 3—severe epithelial lifting, near total distortion of villi, ulceration of mucosa, or disintegration of lamina propria. Scores were based on the highest score observed on three to five sections within the same sample. A score of ≥2 was defined as NEC.

### 2.4. Cytokine Analysis

Cytokine analysis of plasma and ileal homogenate was performed for interleukin (IL)-1β, tumor necrosis factor (TNF)-α, IL-6, IL-12 p70, interferon (IFN)-γ, CXCL1 (C–X–C chemokine ligand 1), and IL-10 with a Luminex-based ProcartaPlex Mouse Cytokine and Chemokine Panel (eBioscience, San Diego, CA, USA), per the manufacturer’s instructions. Small intestinal samples were homogenized using a bead beater (Next Advance INC, Troy, NY, USA) in a buffer containing phosphatase and protease inhibitors (Millipore, Burlington, MA, USA) and phenylmethanesulfonylfluoride (Sigma-Aldrich). Samples were run in duplicate on a BioPlex 200 (Bio-Rad, Hercules, CA, USA), and results were calculated on the basis of a seven-point, five-parameter logistic standard curve for each analyte. Final cytokine levels were normalized to total protein concentration (mg/mL) and logarithmically transformed using log(x) (or log(x + 1) when values were <1) to reduce skew in data.

### 2.5. Microbiome Analysis via 16S Ribosomal RNA (rRNA) Sequencing

The Microbiome and Transcriptomics Core at OUHSC performed pup cecal bacterial composition analyses. Following euthanasia, stool samples from 48 pups were collected in Stool DNA Isolation Kit (Norgen Biotek Corp., Thorold, ON, Canada) bead tubes. Samples were immediately snap-frozen in liquid nitrogen and stored at −80 °C until bacterial extraction, performed per manufacturer’s instructions. A NanoDrop Lite Spectrophotometer (ThermoFisher) was used to evaluate and quantify isolated DNA. An Illumina kit was used for PCR amplification of the variable V3 and V4 regions of the 16S rRNA gene. Libraries of 300 bp reads were sequenced over 600 cycles on the Illumina MiSeq platform using the MiSeq Reagent Kit v3. The microbiome bioinformatics platform, QIIME 2, was used to analyze the 18 Gb of data [35]. Amplicon sequence variants (ASVs) were evaluated in comparison with Greengenes, a 16S rRNA reference database [36]. PERMANOVA was employed to determine the β-diversity distances within and between groups, and statistical significance of α-diversity was determined by Kruskal–Wallis test. Phylogenetic Investigation of Communities by Reconstruction of Unobserved States 2 (PICRUSt2) analysis was performed from enzyme commission (EC) numbers grouped into MetaCyc reactions to identify bacteria-affected metabolic pathways [37]. A nonparametric permutation test was used to calculate α-diversity on the basis of differential abundance and PICRUSt values, and taxonomic count data were evaluated using negative binomial distribution. The *p*-values were adjusted using false discovery rate (FDR).

### 2.6. Ileal RNA-Seq Profiling and Enrichment Analysis

Tissues were preserved in RNAlater (Invitrogen, Carlsbad, CA, USA) per the manufacturer’s instructions and stored until analysis at −80 °C. Following homogenization via QIAshredder (Qiagen, Germantown, MD, USA), RNA was isolated using the RNeasy Plus Mini Kit (Qiagen) following the manufacturer’s instructions and quantified using a NanoDrop Lite Spectrophotometer. cDNA reverse transcription was achieved using a High-Capacity cDNA Reverse Transcription Kit (Applied Biosystems, Foster City, CA, USA) and 1 µg of RNA. Following established protocols, the CORALL Total RNA-Seq Library Prep Kit v2 (Lexogen, Greenland, NH, USA) was used to create stranded RNA-Seq libraries for 3–5 animals per group. Each library was constructed using 200–500 ng of RNA and indexed for multiplexing. Samples were normalized, and eight libraries were pooled and run on the Illumina NovaSeq 6000 Platform (San Diego, CA, USA) at the Oklahoma Medical Research Foundation Clinical Genomics Center. A custom computational pipeline, including the open-source gSNAP, Cufflinks, and R for sequence alignment and ascertainment of differential gene expression [38], was used to analyze sequencing. gSNAP [39] was used to map generated reads to the mouse genome (mm10), and expression (fragments per kilobase of transcript per million mapped reads, FPKM) derived by Cufflinks [40] and differential expression were analyzed via ANOVA (analysis of variance) in R.

The log_2_ fold change of genes significant at an FDR < 0.05 were submitted for analysis by Ingenuity Pathway Analysis (IPA) software (Qiagen) to identify biological functions and pathways of interest using the Ingenuity Knowledge Base repository (Ingenuity Systems, Inc., Redwood City, CA, USA). Fisher’s exact test was used to calculate a *p*-value determining the probability of overlap between gene enrichment in the dataset and that of the reference set. To assess the activation state of a pathway and upstream regulators, the consistency of the match between the observed and predicted pattern was calculated (Z-score). A positive Z-score signifies a regulator or pathway as activated, while a negative Z-score indicates inhibition. Canonical pathway analysis was used to identify networks from the IPA library most significantly modulated across both groups. The significance of the association between each dataset and the canonical pathway was measured as a ratio of the number of genes of interest relative to the total number of genes annotated in Ingenuity Pathway knowledge base functional pathways.

### 2.7. Statistical Analysis

GraphPad Prism version 9 (GraphPad Software, La Jolla, CA, USA) was used for statistical analysis. Survival curves to assess mortality were obtained via Kaplan–Meier survival analysis. Data are presented as the mean ± standard error of the mean (SEM), and specific sample sizes are denoted in figure captions. Unless otherwise noted, data were analyzed by one-way ANOVA (including Tukey’s multiple comparison analysis), unpaired Student’s *t*-tests, or Mann–Whitney U test, as appropriate. A *p*-value < 0.05 was considered significant.

## 3. Results

### 3.1. HA35 Protects against NEC Induced by Formula Feeding and Dithizone

**Figure 1:**
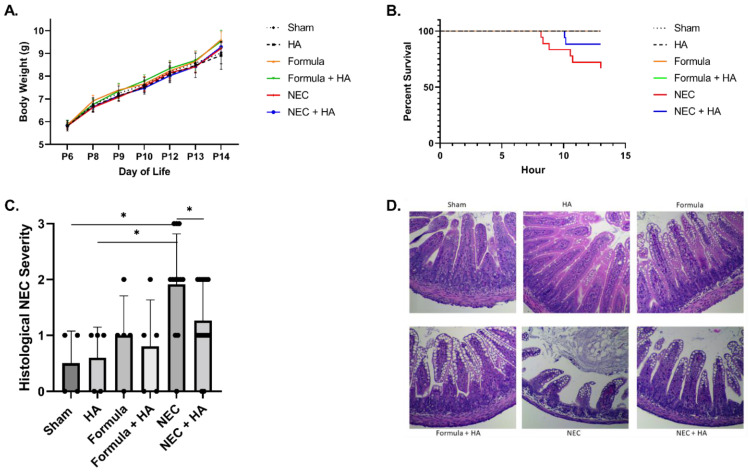
Pretreatment with oral HA35 for 7 days protects against NEC morbidity and mortality. (**A**) HA35 did not affect weight gain among treatment groups (*p* = 0.9972; *n* ≥ 5/group). (**B**) HA35 treatment trended toward increased survival during the 13 h experiment, from approximately 67% (untreated NEC; *n* = 18) to 88% (NEC + HA; *n* = 17; *p* = 0.1209). (**C**) HA35 reduced intestinal histological injury compared to untreated NEC (*n* = 12 (NEC) and 15 (NEC + HA); 1.917 vs. 1.297; *p* = 0.0452). (**D**) Representative histology of sham, HA, formula, formula + HA, NEC, and NEC + HA pups. Data are presented as the mean ± SEM, * *p* < 0.05. HA: hyaluronic acid; NEC: necrotizing enterocolitis; SEM: standard error of the mean.

Our prior studies demonstrated HA35 protects against a murine model of NEC involving Paneth cell dysfunction and the induction of dysbiosis through oral gavage of *Klebsiella* [15]. Here, we tested the potential of HA35 to protect against a secondary mouse model of NEC, replacing dysbiosis with another common risk factor for the development of NEC, formula feeding [18]. HA35 treatment for 7 days did not affect pup weight gain (Figure 1A). The combination of enteral formula and Paneth cell dysfunction induced NEC-like injury in pups, but NEC pups pretreated with HA35 trended toward increased survival, from approximately 67% (untreated) to 88% (NEC + HA) (*p* = 0.1209; Figure 1B). Treatment with HA35 also significantly decreased histological injury scores in comparison to untreated NEC pups, from an average of nearly 2 (untreated NEC) to approximately 1.27 (NEC + HA) (Figure 1C,D). To determine whether a reduction in local or systemic inflammation might be responsible for the reduced histological injury and trend of increased survival, we measured plasma and tissue cytokine levels of IL-1β, TNF-α, IL-6, IL-12 p70, IFN-γ, CXCL1, and IL-10. Although these cytokines are representative of those altered during human NEC [41], no significant differences among groups were noted (Supplementary Figure S1).

### 3.2. Effects of Oral HA35 on Intestinal Microbiome Taxonomy and Functional Prediction

**Figure 2:**
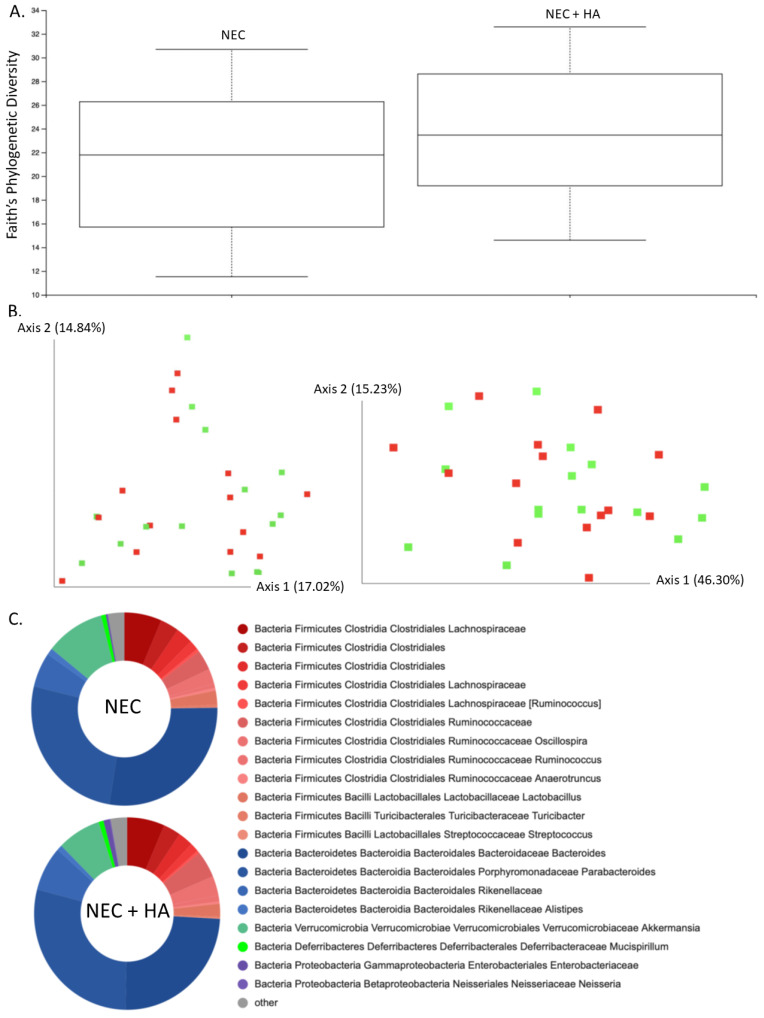
Cecal microbiome composition analysis comparing NEC and NEC + HA pups. (**A**) Faith’s phylogenetic diversity metric representing species richness within mouse pup microbiomes differentiated by treatment (*n* = 29; Kruskal–Wallis *p* = 0.295). (**B**) PCoA plot of Bray–Curtis (**left**) and Weighted Unifrac (**right**) beta diversity matrices (NEC = red, NEC + HA = green). Percentage confidence values for each distance matrix displayed on axes in two dimensions (PERMANOVA: F = 1.065 and *p* = 0.379 (Bray–Curtis); F = 0.589 and *p* = 0.641 (Weighted Unifrac). (**C**) Taxonomy plots using a naïve Bayes classifier trained on the most recent Greengenes 16S rRNA database, with ASV reads taxonomically classified and filtered for genera > 0.5% of total microbiome composition for any one sample. Genera falling below the 0.5% mark were placed in “other” (gray). NEC: necrotizing enterocolitis; HA: hyaluronic acid; PCoA: principal coordinates analysis; ASV: amplicon sequence variance.

In order to ascertain a potential role of the microbiome in HA-induced protection from NEC, stool samples were collected following euthanasia, and the microbial composition was characterized. No statistically significant differences were noted in the microbiota alpha diversity of NEC compared with NEC + HA pups (Figure 2A; Kruskal–Wallis *p* = 0.295) or among all pup groups (Supplementary Figure S2A; Kruskal–Wallis *p* = 0.794). Bray–Curtis and Weighted Unifrac beta diversity matrices for NEC compared with NEC + HA pups (Figure 2B; PERMANOVA: F = 1.065 and *p* = 0.379 (Bray–Curtis) and F = 0.589 and *p* = 0.641 (Weighted Unifrac))) and for all pup groups (Supplementary Figure S2B; PERMANOVA: F = 1.07 and *p* = 0.348 (Bray–Curtis); F = 1.00 and *p* = 0.43 (Weighted Unifrac)) were plotted using principal coordinates analysis (PCoA) and showed insignificant clustering by group. Unsurprisingly, the gut microbiome of all groups was heavily dominated by Firmicutes and Bacteroides phyla (Figure 2C; Supplementary Figure S2C).

Linear discriminant analysis (LDA) effect size (LEfSe) comparisons revealed no significant differences between NEC and NEC + HA phylum-, class-, family-, genus-, or species-level microbial populations, or among taxonomic groups among all pups (Supplementary Figure S3A,B—groups shown are only those with uniquely associated bacteria; LDA score ≥ 2). No unique bacteria were able to differentiate one pup group from others. Gene content-based clustering of microbial communities did not differ among groups (Supplementary Figure S3C; PERMANOVA: F = 0.907 and *p* = 0.583), and LEfSe analysis of PICRUSt2 output did not suggest many differences in predicted biological pathway enrichment among groups (Supplementary Figure S3D; LDA score ≥ 2).

### 3.3. Effect of Oral HA35 on Ileal RNA-Seq Transcriptional Profiles

**Figure 3:**
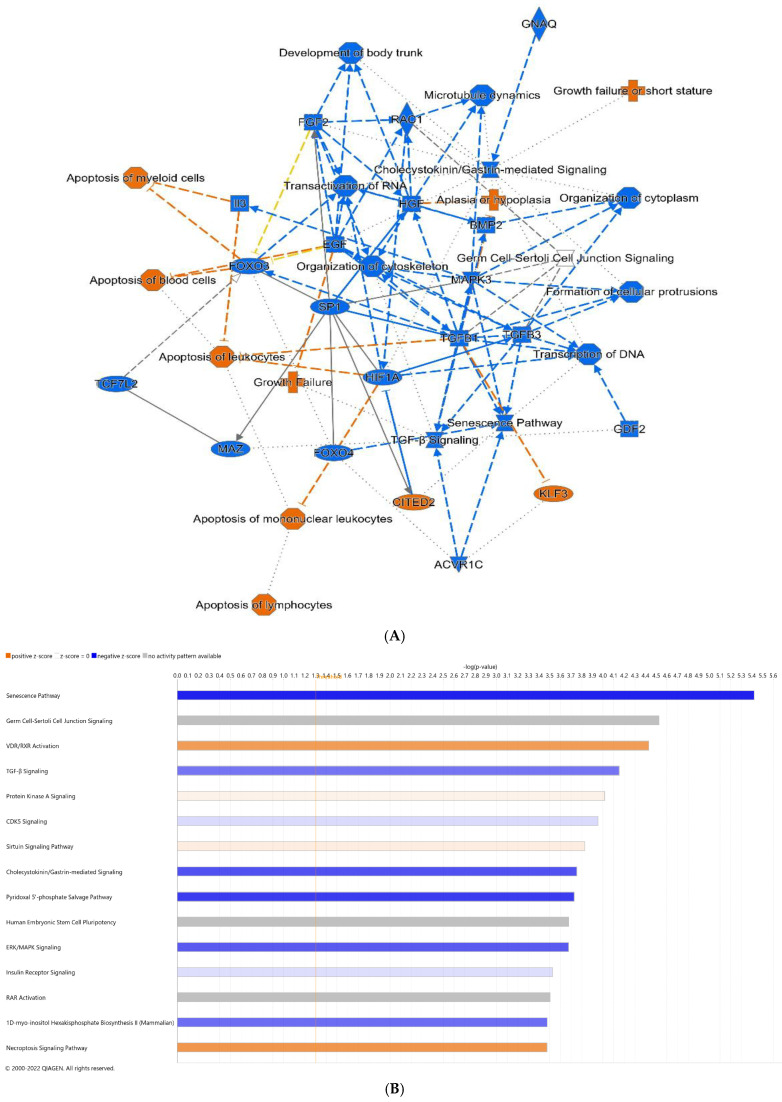
Differential gene expression of terminal ileum of formula pups compared with shams. (**A**) Predicted graphical summary of differentially regulated pathways and genes. (**B**) Top 15 predicted differentially regulated canonical pathways. Blue = Z-score < −2 (downregulation), orange = Z-score > 2 (upregulation), scaled to activation level (light = low; dark = high). White = Z-score close to 0. Gray = fewer than four molecules in dataset, resulting in no prediction.

Given a lack of noted differences in the microbiome, we performed RNA-Seq analysis of distal ileal tissues from pups in the sham, formula, NEC, and NEC + HA groups. As this is the first transcriptomic study of mice subjected to the D/FF NEC model, we characterized the sequential contributions of formula (formula vs. sham), dithizone-induced Paneth cell dysfunction (NEC vs. formula), and HA (NEC + HA vs. NEC) to differential expression of genes (DEGs). Using a significance cutoff of *p* ≤ 0.05, a total of 968 DEGs (303 upregulated, 665 downregulated) were identified comparing formula pups to shams, 35 DEGs (24 upregulated, 11 downregulated) were identified comparing NEC to formula pups, and 24 DEGs (19 upregulated, 5 downregulated) were identified comparing NEC + HA and NEC pups (Supplementary Table S1). DEGs were uploaded to IPA to predict upstream transcriptional regulators, map transcriptional networks, and to identify key molecules and signaling pathways involved in the pathogenesis of NEC in this model, as well as protective effects emanating from pretreatment of NEC pups with HA35. To determine the effects of formula alone, we compared formula pups to dam-fed shams. The graphical summary of pathways and genes predicted to be significantly altered (Figure 3A) indicated increases in loss of blood and myeloid cells, lymphocytes, and leukocytes, as well as increases in pathways and genes related to low growth (growth failure and hypoplasia). Predicted formula-induced downregulated genes and pathways included many involved in stem cell proliferation and maintenance, including transforming growth factor-beta (TGF-β) signaling, RAC1 (RAC family small GTPase 1), forkhead box O3 (FOXO3), bone morphogenetic protein 2 (BMP2), epidermal growth factor (EGF), hepatocyte growth factor (HGF), and mitogen-activated protein kinase 3 (MAPK3). The top 15 canonical pathways associated with formula feeding, with Z-scores > 2 or <−2, are shown in Figure 3B. These included an increase in necroptosis signaling and vitamin D receptor/retinoid X receptor (VDR/RXR) activation, a decrease in cholecystokinin (CCK) and gastrin-mediated signaling, and a decrease in the senescence pathway. Among the most highly increased differentially expressed genes in formula pups were TNF-α and BCAT1 (branched chain amino acid transaminase 1), a gene associated with activated macrophages in inflammatory diseases [42].

**Figure 4:**
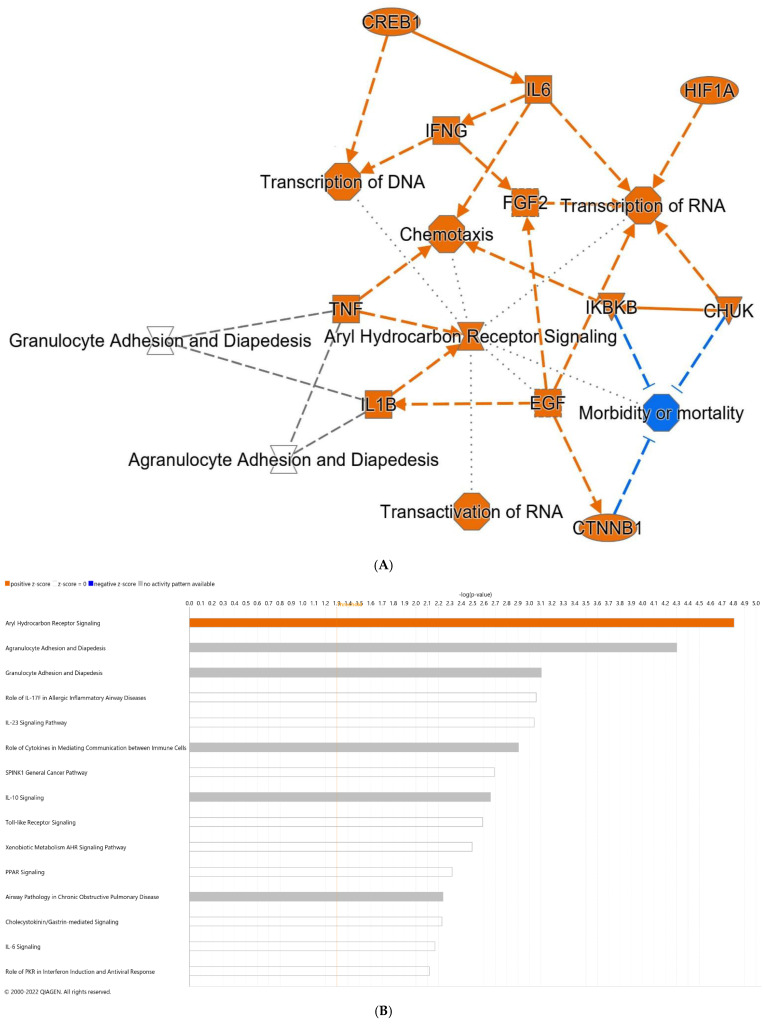
Differential gene expression of terminal ileum of NEC pups compared with pups fed formula alone. (**A**) Predicted graphical summary of differentially regulated pathways and genes. (**B**) Top 15 predicted differentially regulated canonical pathways. Blue = Z-score < −2 (downregulation), orange = Z-score > 2 (upregulation), scaled to activation level (light = low; dark = high). White = Z-score close to 0. Gray = fewer than four molecules in dataset, resulting in no prediction. NEC: necrotizing enterocolitis.

DEGs and alterations in pathways resulting from the addition of dithizone to formula (thereby completing the NEC model), in comparison to formula alone pups, include an increase in IL-6, hypoxia inducible factor 1 alpha (HIF-1α), TNF, IL-1β, and catenin beta 1 (CTNNB1), representing a number of strongly inflammatory mediators (Figure 4A). Among the top predicted canonical pathways were aryl hydrocarbon receptor (Ahr) and Toll-like receptor (TLR) signaling (Figure 4B). Individual genes with the greatest upregulation in expression, comparing NEC to formula pups, included IL-22 and CXCL5 (C-X-C motif ligand 5), an activator of neutrophils [43], while reductions in FCGBP (Fc gamma binding protein) and ST6GALNAC1 (ST6 *N*-acetylgalactosaminide alpha-2,6-sialytransferase 1), genes both associated with goblet cells, were noted.

**Figure 5:**
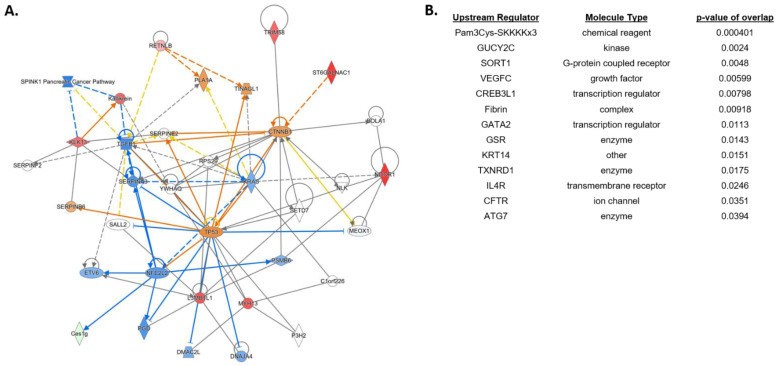
Differential gene expression of terminal ileum of NEC + HA pups compared with untreated NEC pups. (**A**) Predicted network involving differentially regulated pathways and genes. (**B**) Top predicted upstream regulators associated with HA pretreatment of NEC pups. Blue = Z-score < −2 (predicted downregulation), orange = Z-score > 2 (predicted upregulation), red = actual upregulated gene, green = actual downregulated gene. NEC: necrotizing enterocolitis; HA = hyaluronic acid.

Lastly, we compared differentially expressed genes and pathways between NEC pups pretreated with HA35 for 7 days and untreated NEC pups (Figure 5). No canonical pathways met our Z-score threshold, but multiple individual genes of interest were differentially expressed. TRIM58 (tripartite motif containing 58), a negative regulator of TLR2 signaling in myeloid cells with a purported role in reducing intestinal mucosal inflammation [44], was upregulated approximately 16-fold in HA-treated NEC pups. ST6GALNAC1, significantly downregulated in NEC pups compared to formula pups, was upregulated 21-fold in HA-treated NEC pups compared to untreated NEC, potentially indicating the ability of HA35 to reverse components of dithizone-associated pathology. Lastly, resistin-like beta (RETNLB), a goblet cell gene regulating susceptibility to intestinal inflammation through promotion of intestinal barrier function [45], was highly upregulated in HA-treated NEC compared to untreated NEC pups. Upstream regulators predicted to overlap with DEGs in HA-treated NEC pups included, among others, GUCY2C (guanylyl cyclase C), a regulator of intestinal barrier integrity [46], and TXNRD1 (thioredoxin reductase 1), an antioxidant scavenger associated with attenuation of intestinal barrier injury [47].

## 4. Discussion

Human milk is protective against NEC [6,7], but the relative importance of the innumerable compositional differences between HM and formula, in relation to NEC risk specifically, remains unknown. Most studies attempting to define the protective mechanisms of HM focus on the components of HM that are absent in formula (e.g., [15,48,49]), rather than the possibility of formula, itself, inducing pathogenesis. Broad observations in the 1970s suggested hyperosmolar feeds, such as those provided by elemental infant formula, may cause NEC [19,20], inducing a consensus statement by the American Academy of Pediatrics (AAP) that osmolarity of infant formula should not exceed 400 mOsm/L (roughly equivalent to osmolality of 450 mOsm/kg H_2_O) [50]. While the standard of care today still utilizes 450 mOsm/kg H_2_O as the cutoff for formula osmolality, nutritional fortifiers, supplements, and medications taken in conjunction with or near the time of feeding likely result in the breaching of this osmolality threshold [51].

Nutritional studies across a variety of species utilizing hypertonic solutions in the intestine have indicated mixed results on the potential pathogenicity of these solutions to the epithelium [52,53,54,55], but few studies have looked at hypertonicity in the context of NEC development. Miyake et al. evaluated the effects of 849 mOsm/kg H_2_O and 325 mOsm/kg H_2_O formula, in combination with hypoxia and lipopolysaccharide administration, on inflammation, histological injury, and incidence of NEC in P9 mouse pups, but neither intestinal injury nor NEC incidence was found to be associated with formula osmolality [56]. However, Chen et al. evaluated the ability of formula to induce hypoxia in the neonatal intestine of mice, finding both an upregulation of hypoxia-associated genes and an increase in intestinal mucosal damage in the early neonatal intestine after only a single formula feed [24]. These changes were likely a result of either the osmolarity or the caloric density of the formula. Interestingly, we did not find increased mortality or histological injury in pups fed high-osmolality RMS formula alone. Despite studies indicating that bovine-based infant formula increases baseline intestinal inflammation (IL-8 and IL-1β) compared to HM in the first postnatal month [57], formula pups did not show significantly higher tissue or systemic inflammatory cytokine levels compared to shams.

Rather than an activation of inflammatory pathways (e.g., JNK (c-Jun N-terminal kinase) and ERK/MAPK (extracellular signal-regulated kinase/MAPK)) by hypertonicity [58], our results indicated a downregulation of these pathways in formula mice compared to dam-fed shams, potentially indicating that the formula composition, rather than osmolality, is driving transcriptional differences. Compared with dam-fed shams, pups fed formula in this model were characterized by decreased growth factor signaling (e.g., EGF, HGF, specificity protein 1 (Sp1), and TGF-β (transforming growth factor beta)) related to epithelial proliferation, particularly that of stem cells. Importantly, these same pathways, in part, mediate cellular migration and repair following intestinal injury and are known to be disrupted during NEC [41,59,60]. These growth factors, notably absent in RMS and human infant formula, are present in both HM and amniotic fluid [61]. Fetal swallowing of amniotic fluid containing these growth factors during the third gestational trimester is thought to provide trophic influences on the developing intestine [62], but these growth influences are forfeited with premature birth and frequently not replaced through HM feeds. These proliferative and regenerative pathways are likely downregulated in formula pups due to their absence in RMS formula and, conversely, inclusion in dam-fed milk. While a reduction in growth factor signaling may not be outwardly detrimental in these pups during physiological homeostasis, these growth factor signaling pathways are critical in the response to an intestinal inflammatory insult, such as that provided by dithizone-induced Paneth cell dysfunction.

Comparing ileal transcriptomes of NEC (formula + dithizone) and formula pups, we would expect most differences to relate to the addition of dithizone in the NEC pups. Relatively few genes were differentially expressed between these groups, but a strong inflammatory presence was notable in NEC pups, with a large upregulation of IL-1β and IL-22, two cytokines associated with development of human NEC [63,64]. Interestingly, a signature of reduced goblet cell function was also identified in NEC pups. Paneth and goblet cells, specialized and protective secretory cells of the intestinal epithelium, are reduced in number and function in preterm infants [65,66], and further still in infants developing NEC [67,68]. To induce NEC in this model, we treated formula mouse pups with the zinc chelator, dithizone, purportedly targeting exclusively Paneth cells [69], thereby mimicking the functional loss of Paneth cells noted in human NEC. However, while dithizone does not impact intestinal goblet cell numbers [14], the reduction in goblet cell expression of FCGBP and ST6GALNAC1 genes in NEC compared to formula pups, along with a recent study suggesting that zinc deficiency hinders goblet cell function [70], indicates that dithizone-induced zinc chelation may potentially influence goblet cell function. Expression of FCGBP, a gene coding for the protein to which the prominent goblet cell mucin, Muc2, is bound [71], was significantly downregulated. While the function of Fcgbp has not been fully defined, the protein is thought to play a protective and anti-inflammatory role in immune defense of the intestine [72]. Low levels of Fcgbp, indicating a structural weakening of the mucus layer, have been associated with intestinal diseases such as ulcerative colitis and colorectal cancer [73,74]. The goblet cell gene, ST6GALNAC1, encodes a sialyltransferase producing a truncated *O*-glycan containing a sialyl-Tn (sTn) antigen [75]. Highly expressed in cancer stem cells, ST6GALNAC1 enables cell proliferation, migration, and invasion through upregulated Akt signaling [76]. Interestingly, while the presence of sTn in adult tissue often signals carcinogenesis, sTn glycosylation patterns are common in the fetal intestine, as well as in amniotic fluid, potentially indicating a role in fetal intestinal development [77]. In NEC pups, the reduction in ST6GALNAC1 may, in part, result in the inability of the intestinal epithelium to regenerate and heal in the face of excessive inflammation.

Transcriptomes of NEC pups pretreated with HA, in comparison to untreated NEC, indicated a 21-fold increase in ST6GALNAC1 expression. Given the presence of sTn in amniotic fluid and known effects of HA35 on accelerated development of the intestine [13], increased expression of ST6GALNAC1 and, presumably, its glycan product, sTn, may represent a promotion of intestinal development and maturation in HA-treated NEC pups. In addition, the tolerogenic phenotype dictated by ST6GALNAC1, noted in tumor microenvironments [78] but not yet studied in the developing intestine, may result in a reduction in Th1-induced cytokines and dendritic cell surveillance in HA-treated NEC pups, potentially quelling hyperinflammation in the immature intestine. RETNLB, a gene encoding the goblet cell mucus protein, resistin-like molecule β (RELMβ), was highly upregulated in HA-treated NEC pups. As an antimicrobial protein, RELMβ is released into the intestinal lumen, promoting intestinal barrier function through targeted regulation of Gram-negative bacteria [79]. The reduction in NEC pup mucus integrity induced through the downregulation of FCGBP may be reversed through HA35-induced upregulation of RELMβ, resulting in spatial segregation of potentially pathogenic microbes from the mucus and epithelial barrier. In addition, RELMβ promotes intestinal epithelial cell regeneration following injury via a CD4^+^ T cell-dependent mechanism [80], potentially explaining the reduction in histological injury in HA-treated NEC pups.

Evidence of the importance of HA, both endogenously and exogenously (e.g., HM) supplied, to normal gastrointestinal development is growing. Mouse pups treated with the HA-binding inhibitor, PEP-1, from P7–P14 showed a significant reduction in intestinal stem cell proliferation, crypt height, and villus depth [81]. Our group noted an increase in goblet and Paneth cell numbers, as well as potentially beneficial shifts in microbiome composition, with 7-day HA35 treatment of P7 mouse pups, demonstrating accelerated maturation and differentiation of the intestinal epithelium [13]. In addition, HA35 was protective in the D/K mouse model of NEC, decreasing incidence, severity, and mortality of the disease, largely through maintenance of intestinal barrier function [15]. In this secondary mouse model of NEC, employing the risk factors of hypertonic formula feeding and Paneth cell dysfunction, HA35 treatment reduces histological injury and induces a trend toward reduced pup mortality, potentially through differential regulation of goblet cell genes.

Our study was subject to several limitations. Due to the number of treatment groups, cytokine multiplex analysis was hampered by a relatively small sample size per group, likely resulting in trends rather than significance when several of these same cytokines, via RNA-Seq, showed significant alterations. In addition, most pups fed formula in this study suffered from osmotic diarrhea. Microbiota with a relatively slow replication or a luminal spatial distribution, as opposed to attached to the mucus layer, may have been eliminated from analysis due to the hastened intestinal transit time [82], skewing analysis of the microbiome. These and other confounding variables will be addressed in future studies.

## 5. Conclusions

In summary, HA35 pretreatment of pups subjected to a NEC protocol incorporating dithizone-induced Paneth cell dysfunction and hypertonic formula feeding protected against histological injury and trended toward reduced mortality, likely through differential genetic regulation of goblet cell function and innate immune response. Further inquiry into the effects of HA35 on goblet cell numbers in this model and, more generally, goblet cell function, is warranted.

## Figures and Tables

**Table 1:** Rodent milk substitute (RMS) composition (modified from [28]).

Formula Component	Amount (g/kg Body Weight)
Evaporated milk	615
Bovine serum albumin	36
Intralipid 20% intravenous fat emulsion	292
Sunflower oil	10
Teklad vitamin mix	4
Supplemental vitamin mixture ^1^	0.56
Salt mixture solution ^2^	10
Non-calcium mineral mixture ^3^	6
Calcium lactate solution (40 g/L)	10
CaCO_3_ solution (40 g/L)	10
CuSO_4_ (30 g/L)	0.57
ZnSO4 (380 g/L)	0.07
Double-distilled water (ddH_2_O)	6

^1^ Riboflavin (16.7 g/kg), niacin (26 g/kg), pyridoxal hydrochloride (13.9 g/kg), myo-inositol (929.4 g/kg), and l-ascorbic acid sodium salt (14 g/kg). ^2^ NaCl (157 g/kg) and KCl (7 g/L) in ddH2O. ^3^ KH_2_PO_4_ (842 g/kg), NaF (0.246 g/kg), Al_2_(SO_4_)_3_·18H_2_O (0.156 g/kg), and MnSO_4_ (0.042 g/kg).

**Table 2:** Experimental Treatments and Durations.

Treatment Group	Feeding Duration/Type	P14 Dithizone (Y/N)
Sham	P7–P13: Water + Dam P14: Saline	N
HA	P7–P13: HA + Dam P14: Saline	N
Formula	P7–P13: Water + Dam P14: RMS	N
Formula + HA	P7–P13: HA + Dam P14: RMS	N
NEC	P7–P13: Water + Dam P14: RMS	Y
NEC + HA	P7–P13: HA + Dam P14: RMS	Y

## Data Availability

The data presented in this study are available on request from the corresponding author.

## Supplementary Materials

The following are available online at https://www.mdpi.com/article/10.3390/nu14091779/s1: Table S1. RNA-Seq data; Figure S1. Plasma and tissue cytokines; Figure S2. Cecal microbiome composition analysis among all pup groups; Figure S3. LEfSe and PICRUSt2 functional analyses among groups.
