# Supplementary figures and images for: Hyaluronic Acid 35 kDa Protects against a Hyperosmotic, Formula Feeding Model of Necrotizing Enterocolitis
Source: Nutrients. 2022 Apr 24;14(9):1779. doi: 10.3390/nu14091779 (PMC9105773; doi:10.3390/nu14091779)

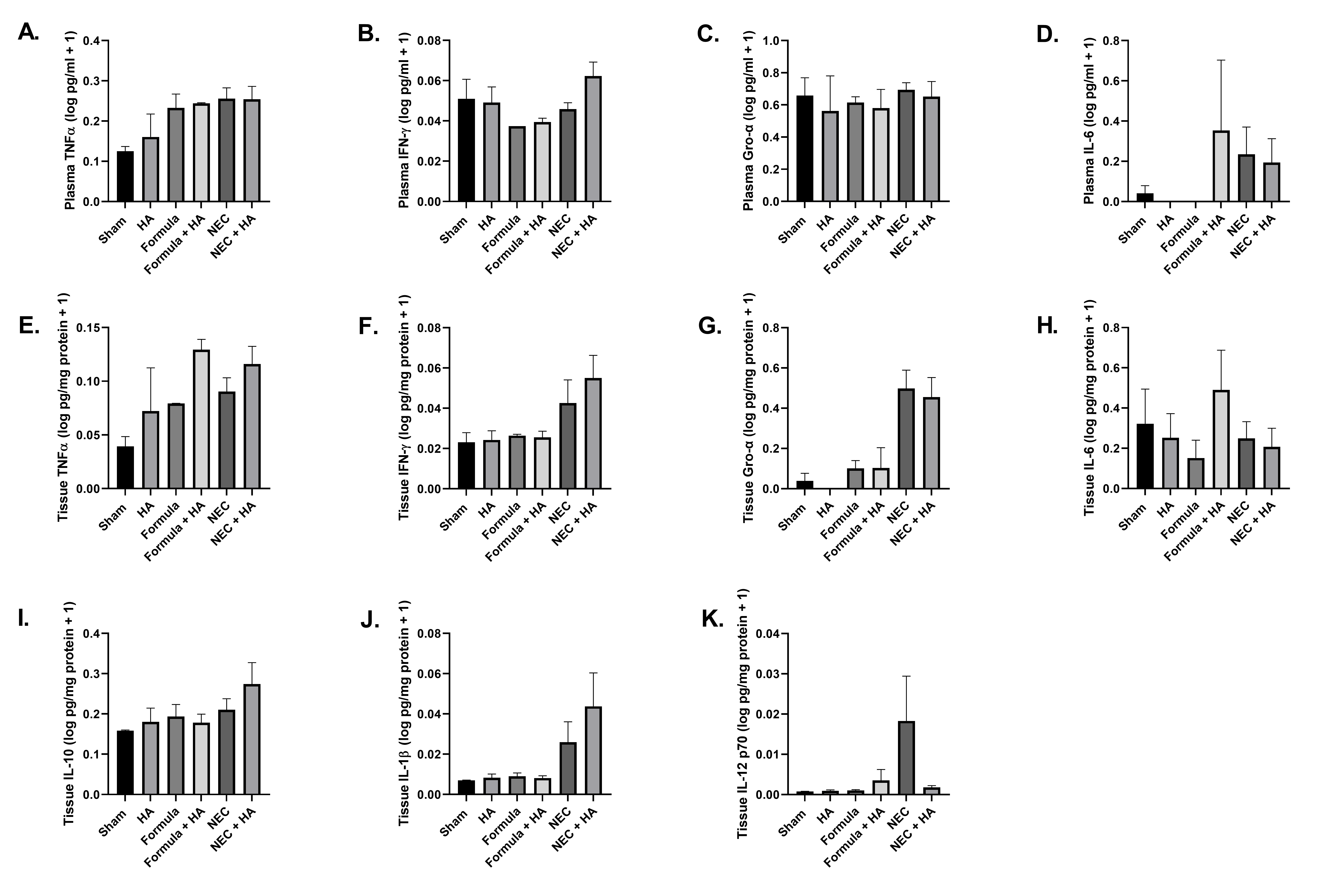

Supplement: Supplementary file 1 [file nutrients-14-01779-s001.zip › Supplementary Figure 1.tif]

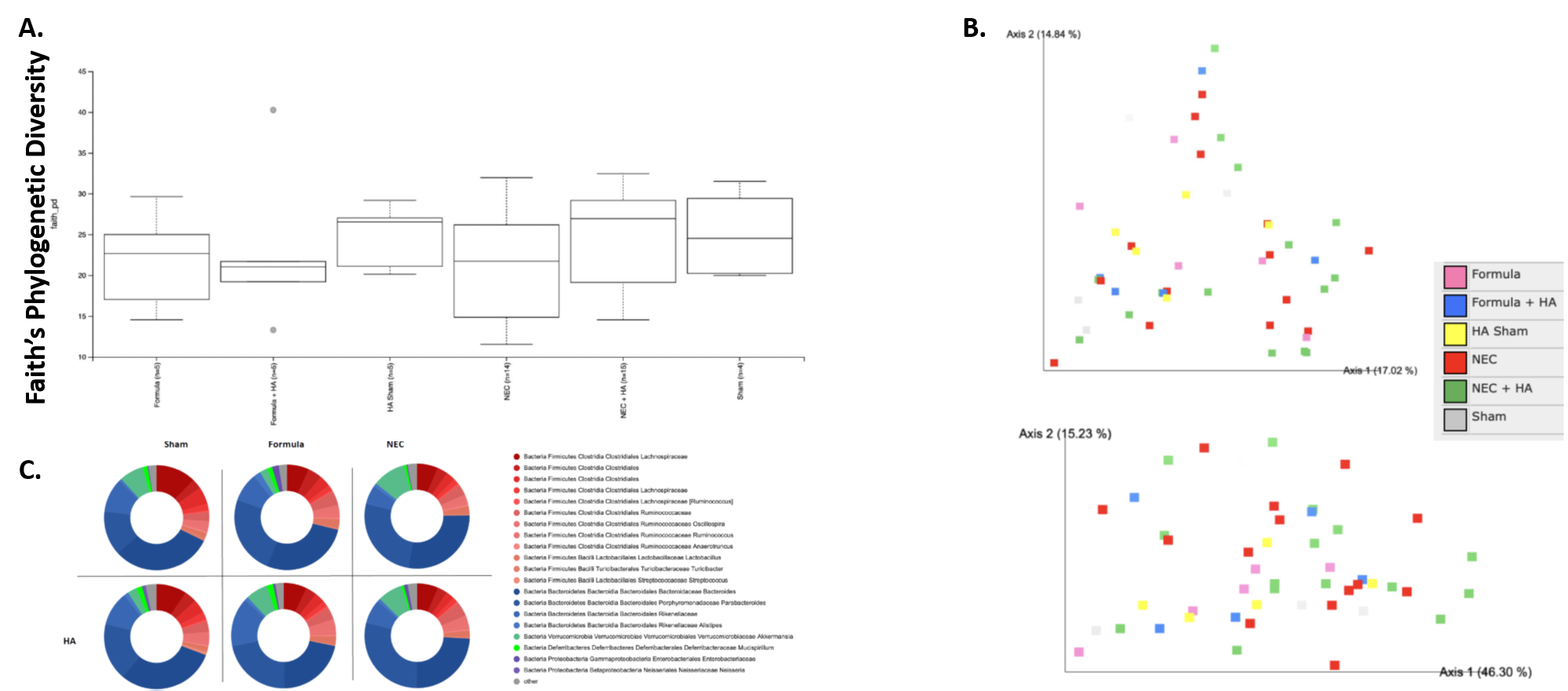

Supplement: Supplementary file 1 [file nutrients-14-01779-s001.zip › Supplementary Figure 2.tif]

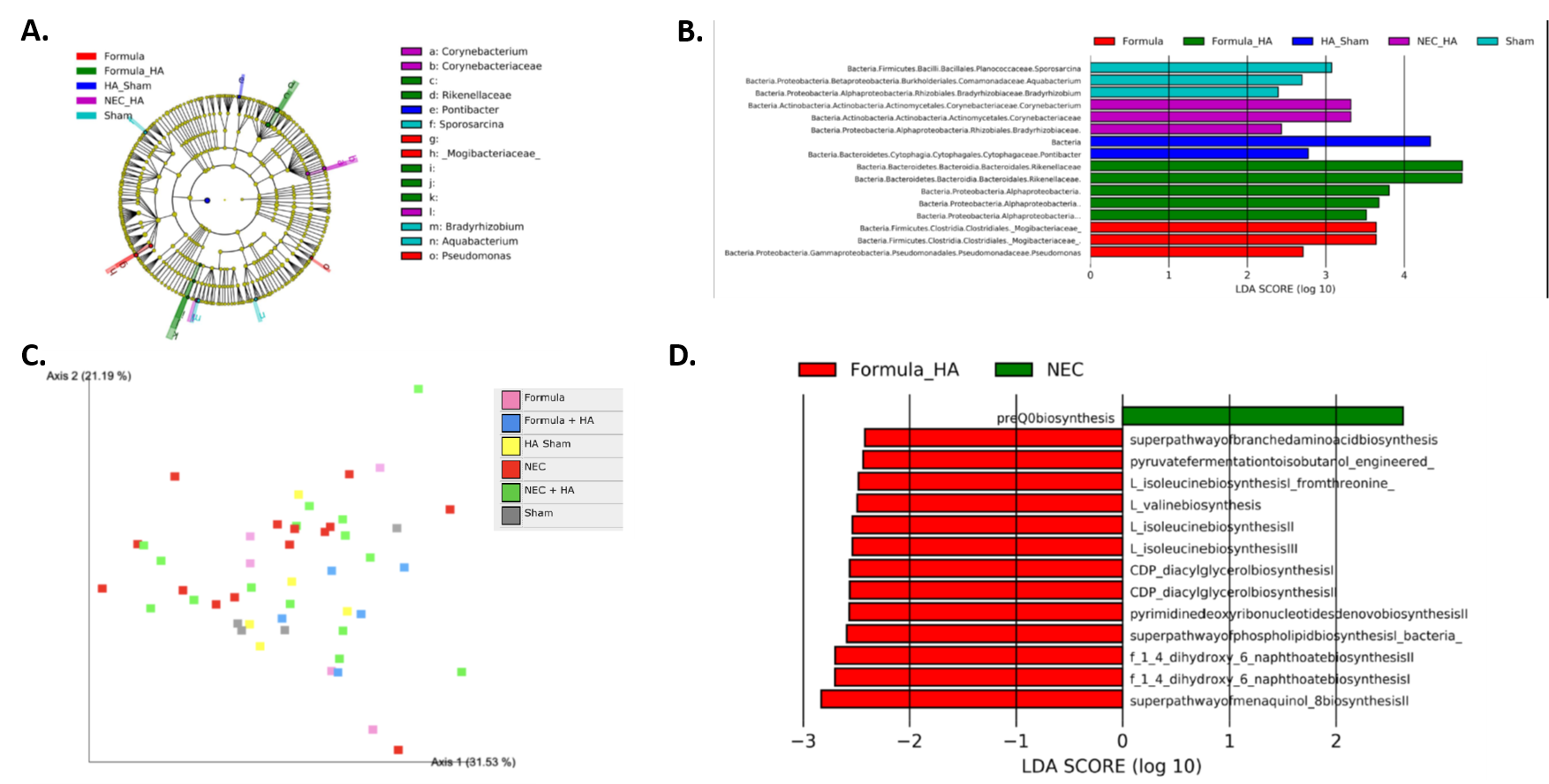

Supplement: Supplementary file 1 [file nutrients-14-01779-s001.zip › Supplementary Figure 3.tif]
